# Record-linkage studies: dates and event-definitions matter hugely

**DOI:** 10.1016/S2468-2667(17)30044-0

**Published:** 2017-03-01

**Authors:** Sheila M Bird

**Affiliations:** MRC Biostatistics Unit, University of Cambridge School of Clinical Medicine and Institute of Public Health, Cambridge CB2 0SR, UK

For 2006–10, Perviz Asaria and colleagues^1^ assessed what proportion of deaths in England with acute myocardial infarction as an underlying or contributing cause were in people admitted to a hospital in the 28 days before death, and whether acute myocardial infarction was one of the recorded diagnoses in such hospital admissions. The authors also wanted to set acute myocardial infarction deaths with a 28 day antecedent of hospital-admission in broader context: that of people admitted to hospital for whom an acute myocardial infarction event was diagnosed. Asaria and colleagues estimated that as many deaths with acute myocardial infarction as underlying cause had occurred without having had a 28-day antecedent hospital admission as had occurred within 28 days of hospitalised acute myocardial infarction-events.

In such a study, dates and definitions matter. In the methods section of their paper, Asaria and colleagues note: “We use the term hospital admission to refer to a continuous spell of care”. Therein lies statistical danger because admission occurs on a specific date, whereas hospitalisation has a variable duration. Moreover, although the date of transition from first to second finished consultant episode (FCE) within a single hospitalisation is recorded by NHS Digital, the date of diagnosis of acute myocardial infarction within the first or subsequent FCE is not.

By contrast, for evaluating Scotland’s National Naloxone Policy,^2^ 28 day look-backs from opioid-related deaths (ORDs) in 2006–2010 were precisely defined: to prison release, with day of prison-release as day 1 so that prison-release-day ORDs were counted;^3^,^4^ and to hospital-discharge with day after hospital-discharge as day 1 so that deaths on or at admission were excluded.^3^,^5^,^6^

Editors should allow authors of record-linkage studies properly to convey in their methods the careful, logical definitions of at-risk periods that must have been explicit in their linkage-protocol; and to do so without short-circuiting. Definitions need to be sufficiently precise that others, internationally, who seek to do validation studies, can replicate the authors’ approach and test deviations in potentially influential respects. How dates and definitions were specified in the linkage-protocol matter as people other than the research team typically prepare the linkage database: as specified in the protocol.

By focusing on deaths during 2006–10 that were registered with Office for National Statistics (ONS) by March 31, 2012, Asaria and colleagues circumvented the problem that late registration of deaths in England and Wales poses for record-linkage study-teams.^7^ However, the confidential inquiries that the investigators propose could be delayed because deaths with a specific underlying cause cannot be sampled until registered with the ONS, which, for inquest-deaths, might be months or years after the date of death.^8^ In Scotland, fact of death must be registered within 8 days of death having been ascertained.^7^
